# Multi-omics analysis of high-grade serous ovarian cancers reveals heterogeneity and distinct immunological patterns based upon clinico-genomic status

**DOI:** 10.1371/journal.pgen.1012355

**Published:** 2026-10-22

**Authors:** Jing Qian, Rania Bassiouni, Yuxin Jin, Seeta Rajpara, Monica Neuman, Varun U. Khetan, Bodour Salhia, David W. Craig, Joseph W. Carlson, Lynda D. Roman, John D. Carpten

**Affiliations:** 1 Department of Cancer Biology, Keck School of Medicine, University of Southern California, Los Angeles, California, United States of America; 2 Department of Integrative Translational Sciences, Beckman Research Institute, City of Hope, Duarte, California, United States of America; 3 Department of Obstetrics and Gynecology, Los Angeles General Medical Center, Los Angeles, California, United States of America; 4 Department of Pathology, City of Hope, Duarte, California, United States of America; 5 Department of Obstetrics and Gynecology, Keck School of Medicine, University of Southern California, Los Angeles, California, United States of America; Brigham and Women’s Hospital, UNITED STATES OF AMERICA

## Abstract

High-grade serous ovarian cancer (HGSOC) is characterized by remarkable intratumoral heterogeneity and variable tissue architectures, dictating differential therapeutic responses. Although *BRCA1/2* mutations correlate with improved responses to DNA-damaging agents, how BRCA genotype shapes the tumor immune microenvironment (TIME) remains incompletely understood. Here, we integrated whole exome sequencing, total RNA sequencing and spatial transcriptomics to profile a cohort of BRCA-mutated (BRCA-MT) and non-BRCA-mutated (BRCA-WT) HGSOC tumors. While bulk transcriptomics highlighted global phenotypic shifts, suggesting a more pro-inflammatory milieu in BRCA-MT tumors, spatially resolved analysis revealed how these differences manifest within specific tissue architectures. Integrated clustering revealed recurrent spatial transcriptional states across genotypes. Specifically, we identified a transcriptionally distinct immune-enriched region (IC7) characterized by low tumor purity, interferon signaling, and antigen-presentation programs. Reference-based RCTD deconvolution provided orthogonal computational support that IC7 was enriched for T/NK and dendritic cells relative to non-IC7 regions. At the patient level, the functional state of IC7 was highly BRCA-dependent: BRCA-MT tumors exhibited stronger enrichment of interferon-α/γ responses and inflammatory signaling than BRCA-WT tumors. Exploratory CellChat analysis further suggested genotype-associated differences in predicted immune-related signaling. We also identify a conserved structural interface between CAF-like region (IC3) and macrophage-rich region (IC4), accompanied by a predicted CXCL12–CXCR4 chemokine recruitment axis. Taken together, these findings support a model in which BRCA mutation status is associated with functional differences within broadly conserved spatial regions, rather than extensive reorganization of the overall spatial architecture. This spatially resolved framework identifies BRCA-associated immune transcriptional patterns in HGSOC and provides a basis for future mechanistic validation.

## Author summary

High-grade serous ovarian cancer is the most common and aggressive form of ovarian cancer. Many tumors carry changes in *BRCA1* or *BRCA2*, genes involved in DNA repair, but it remains unclear how these genetic differences relate to tumor organization and immune activity. This study defines the spatial and molecular features of BRCA-MT and BRCA-WT high-grade serous ovarian cancer using integrated genomic, transcriptomic, and spatial transcriptomic analyses. We found that BRCA-MT and BRCA-WT tumors shared much of their overall tissue architecture. However, immune-related activity differed within specific spatial regions. In particular, BRCA-MT tumors showed stronger interferon and antigen-presentation programs within a recurrent immune-enriched region. Additional computational analyses supported enrichment of immune-cell signals in this region, while a macrophage-rich region appeared more broadly conserved across tumors. These findings suggest that BRCA status may influence the functional immune state of recurrent spatial regions in the tumor microenvironment. Our study provides a spatial framework for future work testing how tumor genetics shapes immune heterogeneity in ovarian cancer.

## Introduction

Ovarian cancer is the second most common gynecologic cancer and ranks fifth in cancer-related deaths among women [1]. Among epithelial ovarian cancer histological subtypes, high-grade serous ovarian cancer (HGSOC) is the most prevalent [2]. Despite advances in treatment, the overall survival rate for HGSOC remains poor, largely due to the recurrence of treatment-resistant disease [3]. HGSOC is characterized by frequent defects in the homologous recombination (HR) DNA repair pathway, often driven by germline mutations in *BRCA1* or *BRCA2*, which occur in 20–22% of cases [4]. Poly-ADP Ribose Polymerase (PARP) inhibitors, such as olaparib, exhibit synthetic lethality with BRCA deficiency, and have been clinically approved for patients with HR-deficient tumors, including those harboring *BRCA1/2* mutations [5]. However, resistance to therapy and disease recurrence remain significant challenges [5,6].

The Cancer Genome Atlas (TCGA) has provided a comprehensive molecular profile of HGSOC, highlighting *TP53* mutations in up to 95% of tumors [4]. BRCA-mutated (BRCA-MT) HGSOCs exhibit elevated somatic mutational burdens and characteristic patterns of genomic instability compared with non-BRCA mutated tumors (BRCA-WT) [4,7]. While genomic instability is known to influence tumor immunogenicity [8], potentially driving differential responses to therapeutic interventions, how BRCA-dependent genomic states specifically shape the spatial organization and communication networks of the tumor immune microenvironment (TIME) remains incompletely understood. To address this, we applied an integrated multi-omics approach to a cohort of HGSOC samples, comparing those with *BRCA1/2* mutations against those without. Through application of whole exome sequencing, bulk RNA sequencing, and spatial transcriptomics, we sought to investigate the spatial distributions, intercellular communication axes, and functional expression programs of malignant, immune and stromal niches driven by BRCA genotype.

## Results

### Genomic characterization of specimen cohorts

To evaluate genomic alterations in our HGSOC cohort (n = 25), we performed somatic whole-exome sequencing (WES) using tumor and matched germline DNA in addition to total RNA-sequencing. Notably, somatic mutation profiles were dominated by mutations in *TP53*, which occurred in 82% of BRCA-MT patients (14/17) and 88% of BRCA-WT patients (7/8) (Fig 1A). *TP53* alterations were predominantly missense mutations (54.5%), and the vast majority of variants (94.7%) were localized between codon 125–300, corresponding to the DNA-binding domain of TP53 [9] (Figs 1B and S1). The most frequently mutated codons were 220 and 273, with variants such as Y220C, R273H, and R273L present in both cohorts. This is consistent with previous reports demonstrating *TP53* mutations to be a hallmark of HGSOC, regardless of BRCA mutation status [10]. Samples without *TP53* mutations in our cohort were subjected to pathology re-review, and all were confirmed to meet the diagnostic criteria for HGSOC.

**Fig 1:**
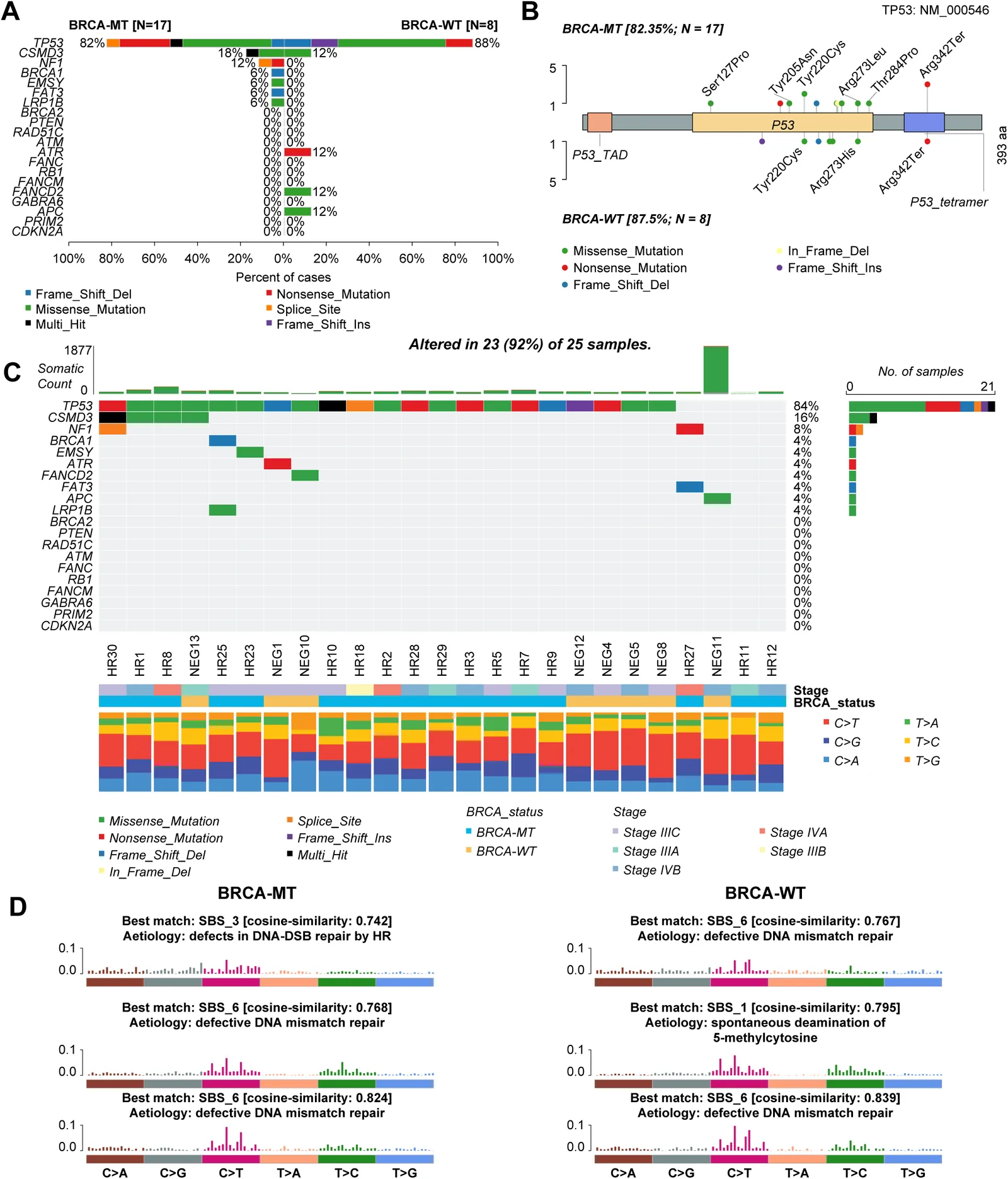
Mutational Landscape of BRCA-MT HGSOC vs. BRCA-WT HGSOC. **(A)** Alteration frequencies of homologous recombination pathway genes in BRCA-MT and BRCA-WT tumors. The horizontal bar plot shows the percentage of cases harboring alterations in each subgroup, and colors indicate the nature of the alteration. **(B)** Lollipop plot mapping identified *TP53* mutations in the BRCA-MT cohort and BRCA-WT cohorts. The y-axis indicates the number of samples harboring the indicated mutations. **(C)** Somatic mutations in 20 genes of interest identified in each patient in our cohort. The top plot displays the somatic mutation count and mutation class distribution in each tumor sample. **(D)** Mutational signature analysis based on single base substitution (SBS) profiles in both groups. Extracted mutational signatures were compared with COSMIC reference signatures using cosine similarity and top three matches are presented here.

Somatic mutations commonly observed in HGSOC, such as alterations in *CSMD3, NF1, FAT3, FANCD2, and EMSY*, were also detected in our cohort (Fig 1C) [4,7]. The frequency and context of other substitutions varied slightly between the BRCA-MT and BRCA-WT groups but were not statistically significant.

We performed mutational signature analysis and refitting to the Catalogue of Somatic Mutations in Cancer (COSMIC) single base-pair substitution (SBS) signatures reflecting mutational processes resulting from known endogenous and exogenous exposures [11]. As defined by COSMIC, BRCA-MT tumors demonstrated a dominant contribution from SBS3, a signature associated with defective DNA double-strand break repair and BRCA dysfunction in cancer genomes [12] (Fig 1D). In contrast, BRCA-WT tumors lacked a prominent SBS3 component and instead exhibited enrichment of clock-like SBS1 and mismatch repair–associated SBS6 [12]. Although SBS6-like contributions were observed in both groups, the presence of SBS3 as a best-matched signature was restricted to BRCA-MT tumors, supporting a BRCA-dependent divergence in underlying mutational processes.

### Genomic instability and homologous recombination deficiency differ by BRCA status

To determine whether genomic instability patterns differ by BRCA mutation status, we performed allele-specific copy number analysis across the BRCA-MT and BRCA-WT samples. We compare these results to 469 BRCA-WT samples from the TCGA cohort (TCGA BRCA-WT). TCGA BRCA-MT samples were not included in the comparative analysis due to the limited number of available cases, which would introduce substantial cohort imbalance and bias genotype-based comparisons. BRCA-MT tumors exhibited a high burden of somatic copy number alterations, characterized by recurrent broad amplifications on chromosomes 1q, 3q, 8q, and 20q, as well as deletions on 4q, 5q, 13q, and 17p (Fig 2A). In contrast, our BRCA-WT cohort displayed a more attenuated genomic profile with fewer large-scale copy number alterations, most notably limited to chromosomes 4q and 13q (Fig 2B). Notably, the TCGA BRCA-WT cohort demonstrated widespread allelic imbalance typical of HGSOC, thereby corroborating the genomic patterns identified in our in-house dataset (Fig 2C).

**Fig 2:**
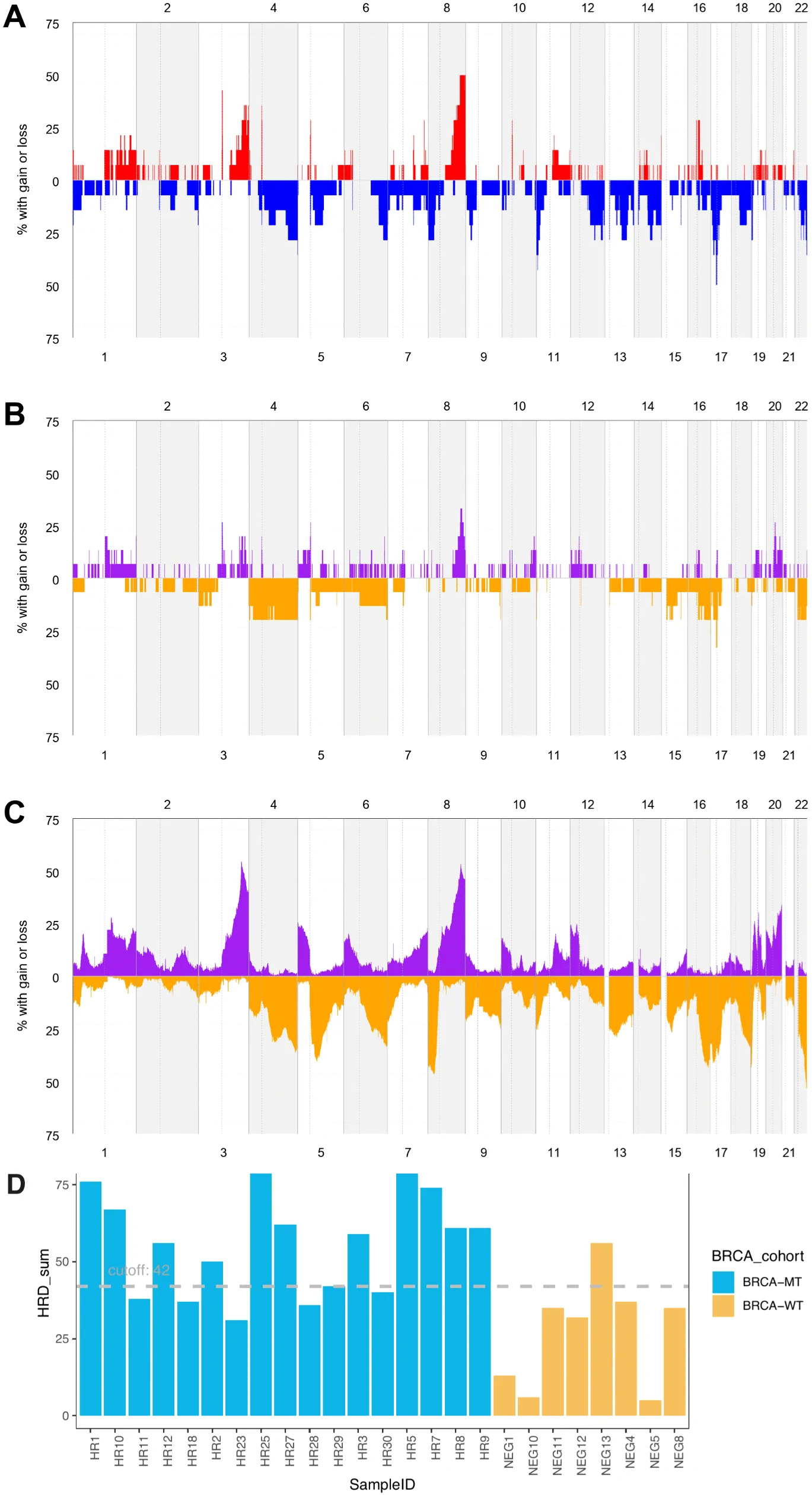
Copy number variation in BRCA-MT, BRCA-WT and TCGA BRCA-WT HGSOC samples. **(A-B)** Copy number variation (CNV) was determined from whole exome sequencing of BRCA-MT (A) and BRCA-WT (B) samples. CNV values are displayed across the genome with thresholds set at −0.5 and 0.5 to define copy number loss and gain. Gains are indicated in red and purple while losses are indicated in blue and orange. **(C)** CNV profiles of BRCA-WT samples from the TCGA cohort. **(D)** Patient-level scarHRD scores in BRCA-MT and BRCA-WT tumors. Individual bars represent independent patients, and the dashed horizontal line indicates the established HRD-score threshold of 42.

To quantitatively assess genomic instability, we calculated HRD scores using scarHRD [13] and stratified the cohort based on the established cutoff of 42 [14] (Fig 2D). The BRCA-MT cohort was largely characterized by high HRD scores, whereas the BRCA-WT cohort was predominantly HR-proficient (median [IQR], 59.0 [40.0–67.0] vs. 33.5 [11.25–35.5]; Hodges–Lehmann location-shift estimate, 28.0; 95% CI, 7.0 to 48.0; Wilcoxon rank-sum p = 0.0015). Notably, the continuous HRD scoring revealed intra-group heterogeneity: a subset of BRCA-WT tumors exhibited robust HR-deficient phenotypes, suggesting that alternative mechanisms of homologous recombination impairment may play a role in these samples.

### BRCA-associated transcriptional programs define distinct proliferative and mesenchymal states

Given the differences in genomic instability and homologous recombination deficiency between groups, we examined whether BRCA status was associated with distinct transcriptional programs using bulk RNA-sequencing data while accounting for treatment status. Treatment-adjusted differential expression analysis retained a clear BRCA-associated transcriptional signal (Fig 3A). Because individual-gene differences can be influenced by sample-specific expression patterns and tumor composition, we next used pre-ranked GSEA to identify coherent molecular programs associated with BRCA status. BRCA-WT tumors were enriched for epithelial-mesenchymal transition, myogenesis, hypoxia and estrogen-response programs, consistent with a more mesenchymal/stromal and stress-adapted transcriptional state (Fig 3B). By contrast, BRCA-MT tumors were enriched for E2F targets, MYC targets, G2M checkpoint, oxidative phosphorylation and DNA-repair-related programs, indicating a more proliferative and metabolically active transcriptional state (Fig 3B).

**Fig 3:**
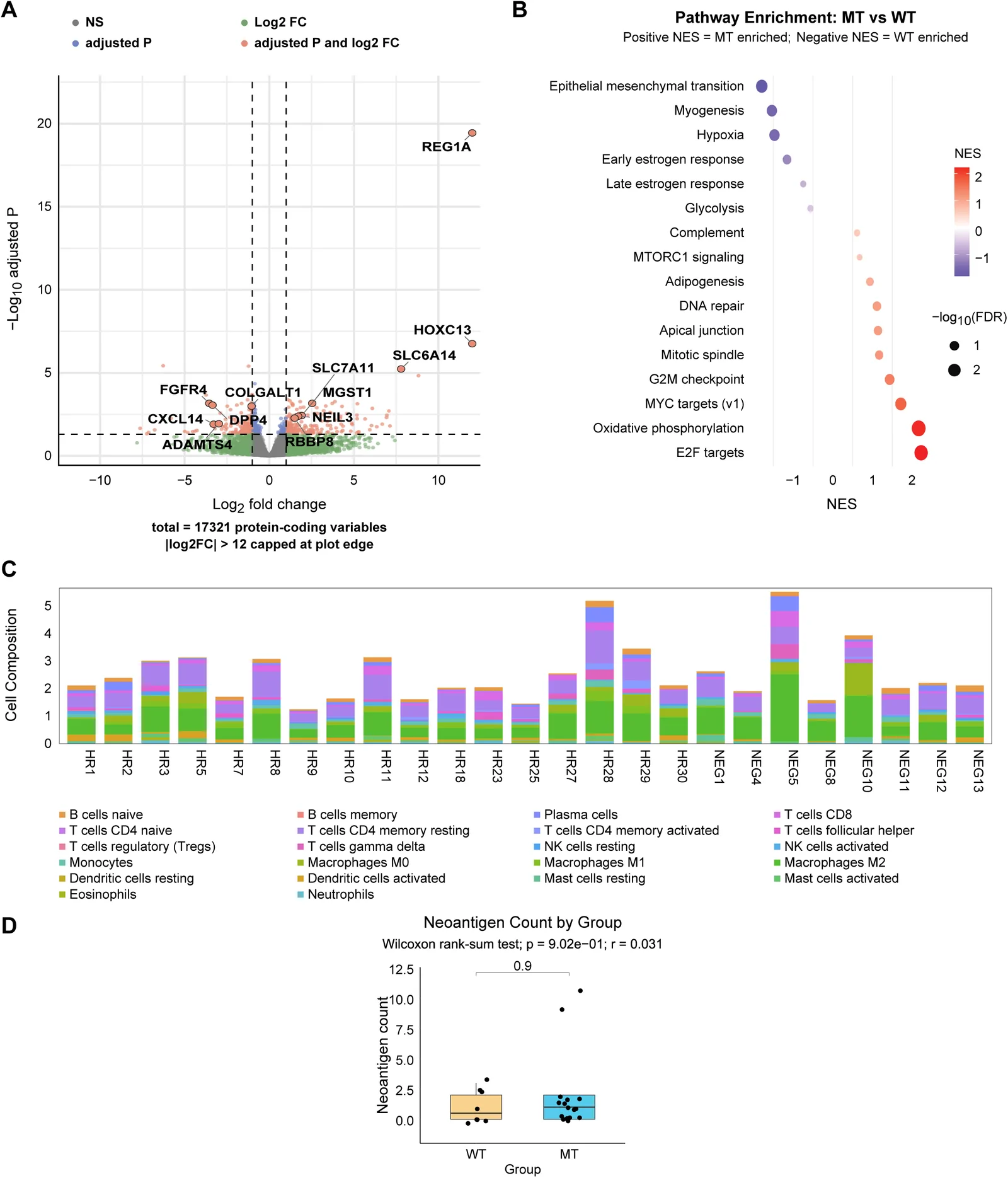
Transcriptional and immune landscape differences between BRCA-MT and BRCA-WT HGSOC. **(A)** Volcano plot showing treatment-adjusted differential gene expression between BRCA-WT and BRCA-MT tumors. The x-axis represents log2 fold change (MT vs WT), and the y-axis represents -log10 adjusted p-value. Genes meeting significance thresholds for both fold change and statistical significance are highlighted. **(B)** Gene set enrichment analysis (GSEA) comparing MT and WT tumors. Each dot represents a Hallmark pathway. The x-axis shows normalized enrichment score (NES; MT vs WT). Dot size corresponds to -log10(FDR), and color indicates direction and magnitude of enrichment. **(C)** Immune cell composition inferred by CIBERSORTx deconvolution across individual samples. Stacked bar plots display the absolute abundance of 22 immune cell subsets per tumor. **(D)** Predicted neoantigen counts by BRCA status. Predicted neoantigen counts across samples grouped by BRCA status are shown in a boxplot. The center line indicates the median for each group, the box bounds represent the interquartile range, and dots represent individual samples.

### Immune composition differs between BRCA-MT and BRCA-WT tumors

Given the distinct transcriptional states associated with BRCA status, we asked whether these differences were accompanied by alterations in the immune cell composition. We applied CIBERSORTx [15] to bulk RNA-seq data to estimate the relative proportions of 22 specified immune cell types. This analysis revealed inter-tumoral heterogeneity across the cohort (Fig 3C), with highly patient-specific immune compositions observed in both BRCA-MT and BRCA-WT groups.

However, this analysis did not identify a clear mutation-status-defined immune polarization pattern. To further evaluate whether BRCA-associated genomic instability translated into increased antigenicity, we quantified predicted neoantigen burden across tumors. Predicted neoantigen counts were not significantly different between BRCA-MT and BRCA-WT tumors (Wilcoxon rank-sum test, p = 0.902; Fig 3D), suggesting that the transcriptional differences associated with BRCA status were not accompanied by a detectable increase in predicted neoantigen burden in this cohort.

### Spatial transcriptomic characterization of individual HGSOC tumors

To define the spatially resolved transcriptomic states of cells within the HGSOC microenvironment, we applied 10x Genomics Visium Spatial Transcriptomic (ST) assay across 48 HGSOC sections from 24 patients, including 17 BRCA-MT and 7 BRCA-WT cases from our study cohort. Following quality assessment as detailed in Methods, we retained 39 sections from 22 patients (16 BRCA-MT and 6 BRCA-WT). On average, each spatial feature was sequenced with approximately 2–4 × 10^8^ total read pairs per sample, achieving high sequencing saturation and robust transcriptome coverage, with a median of ~2,000–6,000 genes detected per feature across samples (S2 Fig). To determine the optimal clustering resolution, we evaluated multiple resolution parameters and examined cluster stability using clustree [16]. The selected resolution represented a balance between cluster granularity and biological interpretability.

### Representative single-sample spatial architecture (HR5)

Resulting analysis of ST data from representative sample HR5, which harbored a germline *BRCA2* mutation, derived seven transcriptionally distinct spatial clusters (Fig 4A and 4B). To identify broad transcriptionally defined cell types within this tumor section, we applied ESTIMATE to normalized feature-level expression data, which revealed concordant spatial patterns of tumor-like, immune-like and stromal-like compartments. Clusters 2, 4, and 7 exhibited higher tumor purity scores, whereas cluster 3 displayed low tumor purity and was enriched for immune-associated features (Fig 4C and 4D). Feature-level ESTIMATE scores were mapped spatially to illustrate tumor, stromal and immune regions across the section (Fig 4E). Stromal scores highlighted extensive fibrotic regions, while tumor purity scores accurately delineated malignant areas (Fig 4E).

**Fig 4:**
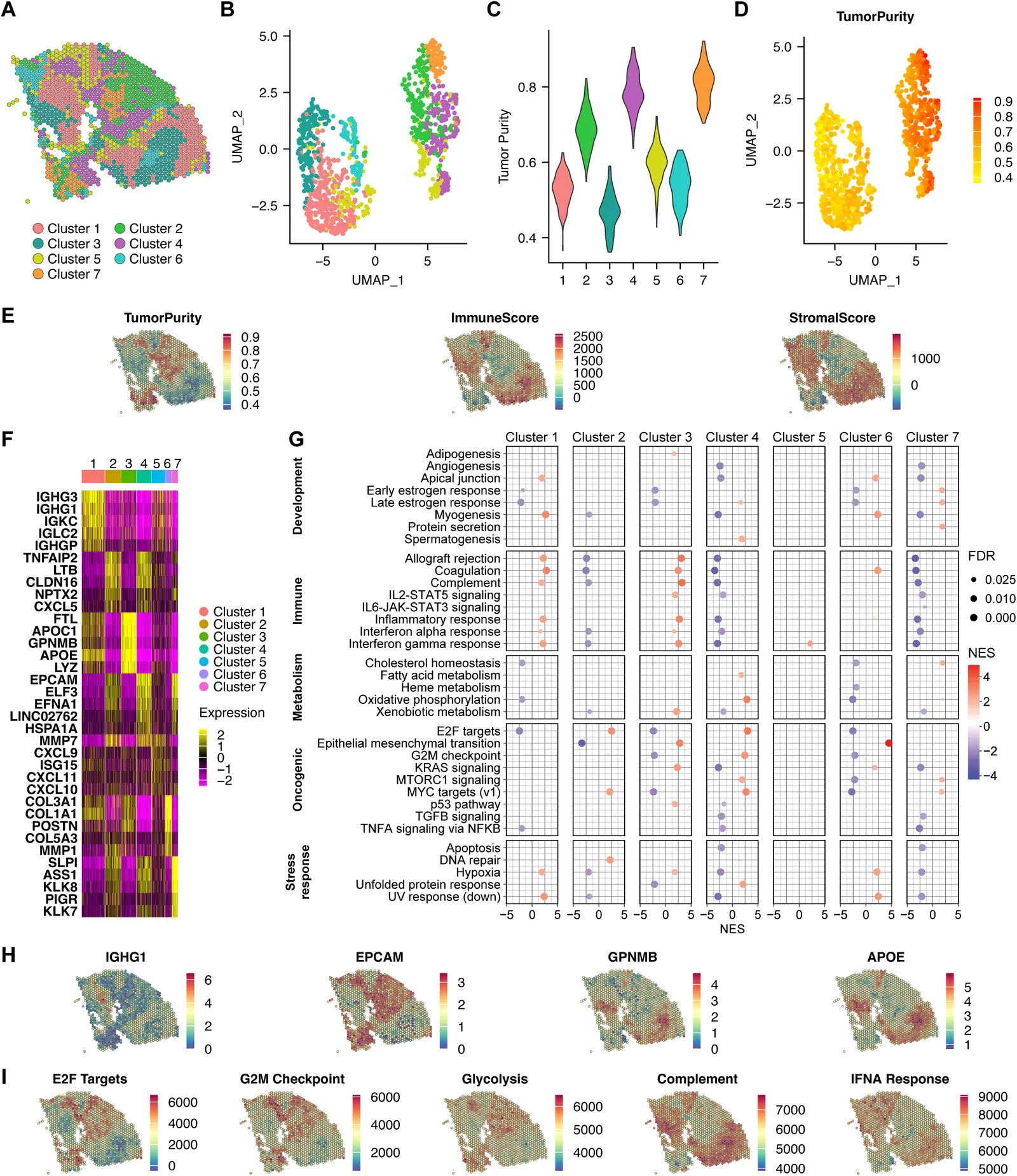
Representative spatial architecture of HGSOC sample HR5. **(A)** Graph-based clustering of sample HR5 identified seven transcriptionally distinct spatial clusters. Each spatial feature is colored according to cluster assignment. **(B)** UMAP projection of sample HR5 colored by cluster identity. **(C)** Violin plot of tumor purity distribution across clusters determined by ESTIMATE. **(D)** UMAP projection colored by tumor purity score. **(E)** Spatial mapping of tumor purity, immune score and stromal score, as determined by ESTIMATE. **(F)** Heatmap of top 5 differentially expressed genes per cluster in sample HR5. Colors represent scaled expression values derived from normalized expression data. Each column corresponds to a single spatial feature, grouped by cluster assignment. **(G)** Cluster-level gene set enrichment analysis (GSEA) against Hallmark gene sets. Only significantly enriched pathways (FDR < 0.05) are shown. Dot size represents –log10(FDR), and color indicates normalized enrichment score (NES). **(H)** Spatial mapping of representative marker genes (e.g., *IGHG1*, *EPCAM*, *GPNMB*, *APOE*) across the tissue section. Color scale indicates normalized gene expression. **(I)** Spatial projection of selected Hallmark pathway scores at the feature level. Color scale represents enrichment score.

Top marker genes defining clusters are depicted in a heat map (Fig 4F). Marker gene expression data analysis further revealed coordinated activation of epithelial, stromal, and immune programs across clusters, consistent with functional compartmentalization within the tumor and immune microenvironment (Fig 4F). To gain insight into the biological processes underlying these spatial compartments, we performed cluster-level gene set enrichment analysis (GSEA). Tumor-dominated clusters were enriched for proliferative and cell cycle–associated pathways, including E2F targets and G2/M checkpoint signaling, whereas immune-enriched clusters exhibited activation of interferon responses, complement signaling, and antigen processing and presentation pathways (Fig 4G).

Spatial mapping of representative marker genes confirmed cluster identities: epithelial tumor markers such as *EPCAM* were enriched in high-purity tumor clusters, whereas immune-associated genes including *GPNMB* localized predominantly to immune-rich regions (Fig 4H). Furthermore, cluster-level functional programs defined by GSEA were also spatially recapitulated when pathway enrichment scores were projected back onto the tissue, demonstrating that transcriptional states identified by clustering reflect localized biological activity within the tumor section (Fig 4I).

### Integrated spatial clustering identifies recurrent tumor, stromal, and immune spatial regions

We next integrated spatial transcriptomic data from all 39 sections to identify recurrent spatio-transcriptional programs across the cohort. Prior to integration, paired sections clustered together, reflecting their shared genetic background, despite moderate intra-tumoral heterogeneity (S3A-S3C Fig). Following integration, genetic background differences were consolidated, allowing us to identify 17 transcriptionally distinct shared integrated clusters (ICs) (Fig 5A). We then mapped integrated clusters back to individual samples to examine their distribution across the cohort. Clusters IC1 through IC7 comprised approximately 97% of all integrated spatial features (Fig 5C) and were consistently present in both BRCA-MT and BRCA-WT tumors (Fig 5D), whereas the remaining clusters (IC8-IC17) were sparsely represented or restricted to a subset of samples. Accordingly, downstream analyses were focused on IC1-IC7, which represent the dominant and shared transcriptional programs across tumors.

**Fig 5:**
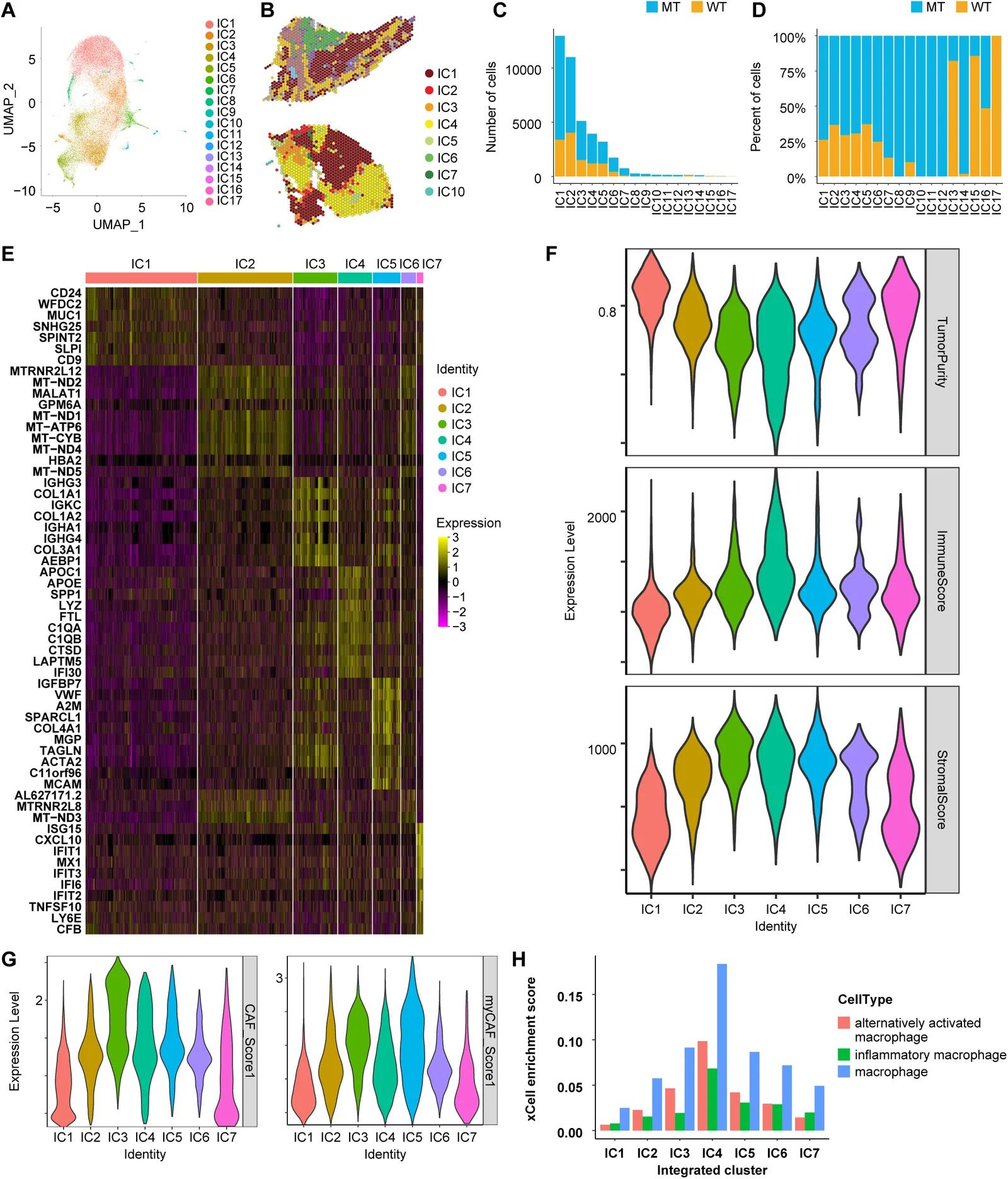
Integrated spatial clustering reveals recurrent tumor, stromal, and immune spatial regions in HGSOC. (A) UMAP embedding of all integrated spatial features (n = 40,002; Visium capture spots) colored by integrated cluster (IC) identity. Seventeen transcriptionally distinct clusters (IC1–IC17) were identified across samples. (B) Representative spatial maps from two tumor sections illustrating the spatial distribution of integrated clusters. Colors correspond to IC identities defined in (A). (C) Number of spatial features assigned to each integrated cluster (IC), stratified by BRCA status (BRCA-MT vs BRCA-WT). (D) The percentage of features in each IC contributed by BRCA-MT and BRCA-WT in the cohort. (E) Heatmap of top differentially expressed genes across integrated clusters (IC1–IC7 shown). Rows represent genes and columns represent spatial features grouped by cluster identity. (F) Violin plots showing distribution of tumor purity, immune score, and stromal score across integrated clusters. Scores were inferred using ESTIMATE. (G) CAF module score distribution across integrated clusters, calculated using Seurat AddModuleScore based on predefined CAF gene signatures. (H) Deconvolution of macrophage-related cell states across integrated clusters (IC1-IC7 shown). Bar plots show relative proportions of inflammatory, alternatively activated, and total macrophage signatures inferred using xCell.

While proportions of IC1 through IC7 varied in individual samples, these clusters were present across all sections, highlighting a conserved core tissue architecture despite inter-tumoral heterogeneity (Figs 5B–5D, S3D–S3E).

Functional annotation of integrated clusters was performed using differential gene expression–based marker identification (Fig 5E), tumor purity estimation (Figs 5F, S4A), CAF module scoring (Fig 5G), immune cell enrichment using xCell (Fig 5H), and Hallmark pathway enrichment analysis (S4B Fig).

IC1 exhibited the highest tumor purity and represented the dominant malignant compartment defined by genes known to be highly expressed in HGSOC including *CD24, WFDC2, and MUC1* (Fig 5E). In contrast, IC3 and IC5 formed stromal-enriched regions characterized by robust expression of canonical extracellular matrix genes, including *COL1A1, COL1A2, COL3A1, DCN, LUM, SPARC,* and *FN1*, along with activated fibroblast markers such as *THBS2* and *CCN2* (Fig 5E). Consistent with this transcriptional profile, CAF module scoring demonstrated strong enrichment of CAF-associated signatures within IC3 and IC5 (Fig 5G). IC7 exhibited enrichment of immune-associated transcriptional programs (Fig 5E). Hallmark pathway analysis further supported these annotations, with IC1 enriched for proliferative programs (e.g., E2F targets, mTORC1 signaling), IC3/IC5 enriched for extracellular matrix and stromal pathways, and IC7 enriched for interferon and immune signaling pathways (S4B Fig).

### Characterization of a shared lipid-associated macrophage spatial transcriptional region in HGSOC

IC4 represented a macrophage-enriched region, displaying elevated expression of canonical tumor associated macrophage (TAM), monocyte, and immune-related marker genes, including *CD68, CD163, GPNMB*, *C1QA, C1QB* and *LYZ.* We used reference-based RCTD deconvolution with a publicly available HGSOC single-cell RNA-seq reference from Zhang et al. (GEO GSE165897) [17], with published annotations collapsed into broad cell-type classes including macrophage, T cell, NK cell, dendritic cell, B cell, CAF, endothelial, tumor/epithelial, mast cell, and mesothelial categories. This analysis supported broad macrophage enrichment in IC4: IC4 regions showed a higher inferred macrophage fraction than non-IC4 regions within the same patients (median 13.86% versus 5.34%; paired Wilcoxon signed-rank p = 4.30 x 10^-5; BH-FDR q = 3.01 x 10^-4; S5C Fig). Notably, this cluster was also strongly enriched in lipid metabolizing marker genes including *APOC1, APOC2*, and *APOE*, indicative of the presence of lipid associated macrophages [18] (LAMs, Figs 5E and 6B). The IC4 LAM compartment was observed in all sections of both BRCA-MT and BRCA-WT tumors and exhibited comparable pathway enrichment patterns across genotypes, indicating that the presence and core functional identity of this macrophage-enriched region are largely BRCA-independent and ubiquitous in HGSOC (Fig 6B).

**Fig 6:**
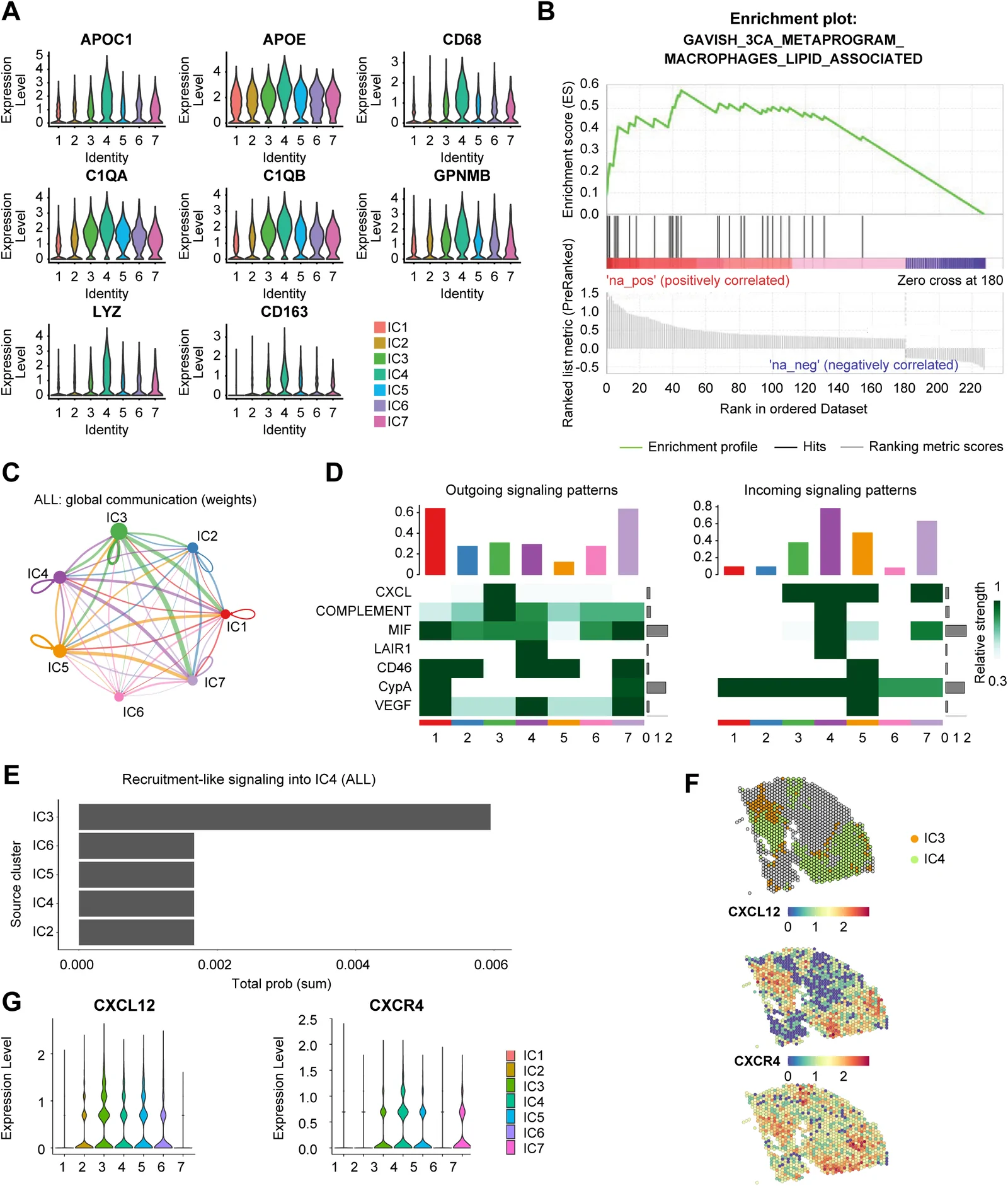
A conserved lipid-associated macrophage-enriched region and predicted CAF-to-LAM communication axis in HGSOC. **(A)** Violin plots showing expression of canonical macrophage markers (*CD68*, *CD163*, *GPNMB*, *LYZ*) and lipid-associated macrophage (LAM) markers (*APOE*, *APOC1*, *C1QA*, *C1QB*) across integrated clusters (IC1–IC7). **(B)** Gene set enrichment analysis (GSEA) demonstrating strong enrichment of a LAM-associated transcriptional program [19] within IC4. **(C)** CellChat-inferred predicted cell–cell communication network across IC1–IC7, computed from pooled integrated-cohort spatial features grouped by IC label. Nodes represent clusters and edge thickness corresponds to interaction strength. **(D)** Predicted outgoing (left) and incoming (right) signaling patterns across selected pathways. Heatmaps display pathway communication strength across clusters. The colored bars on top indicate cluster identities, and the gray bars on the right summarize overall signaling strength for each pathway. CAF-enriched clusters (IC3/IC5) show strong outgoing activity in immunomodulatory pathways (MIF, COMPLEMENT, CXCL), while IC4 displays prominent incoming signaling activity. **(E)** Aggregated recruitment-like signaling strength toward IC4 across source clusters, based on total communication probability. IC3 represents the dominant signaling contributor toward IC4. **(F)** Spatial feature plots showing localization of *CXCL12* expression within CAF-enriched regions and *CXCR4* expression within IC4-enriched macrophage regions, consistent with a stromal–macrophage chemokine interface. **(G)** Violin plots demonstrating elevated *CXCL12* expression in CAF-like clusters (IC3/IC5) and enriched *CXCR4* expression within IC4, supporting a CXCL12–CXCR4–associated predicted communication pattern.

To explore candidate ligand-receptor interactions associated with the LAM-enriched region, we interrogated predicted intercellular communication patterns on pooled integrated-cohort spatial features using CellChat [20]. The predicted global signaling network analysis suggested that stromal-enriched CAF clusters IC3 and IC5 functioned as dominant signaling sources toward IC4 (Fig 6C). Predicted immunomodulatory pathways such as MIF, APP, and COMPLEMENT were enriched in CAF-to-LAM signaling (Fig 6D), raising the possibility that stromal-enriched region may contribute not only to macrophage localization but also to macrophage functional states. Quantification of total recruitment-like signaling into IC4 further identified that IC3 constituted the dominant signaling source (Fig 6E). Consistent with a chemokine-mediated recruitment model [21], *CXCL12* expression was enriched within CAF-like clusters (IC3 and IC5), whereas its cognate receptor *CXCR4* was preferentially expressed in the LAM IC4 cluster (Fig 6F).

### IC7 represents an immune-activated spatial region with BRCA-dependent functional states

IC7 was characterized by strong activation of immune-related pathways, including interferon-α/γ responses, complement activation, and inflammatory signaling, defining IC7 as an immune-enriched region within the HGSOC tumor and immune microenvironment (Fig 7A). Across all shared integrated clusters, IC7 showed the highest patient-level ssGSEA scores for IFN-gamma response, IFN-α response, and TNFα/NF-κB signaling, with inflammatory response also among the highest-ranked signatures (S5A Fig). GSEA further revealed marked BRCA-dependent differences in the functional programs active within IC7. In BRCA-MT tumors, IC7 exhibited significant enrichment of immune-activating pathways, including interferon-α and interferon-γ responses, antigen processing and presentation, and inflammatory signaling, consistent with an immune-engaged transcriptional state. In contrast, IC7 in BRCA-WT tumors exhibited reduced enrichment of these immune pathways and relative enrichment of programs associated with epithelial–mesenchymal transition and tissue remodeling (Fig 7B). The results of our integrated analysis support the localization of a BRCA genotype-associated immune compartment defined by antigen presentation machinery and interferon signaling within the HGSOC tumor architecture.

**Fig 7:**
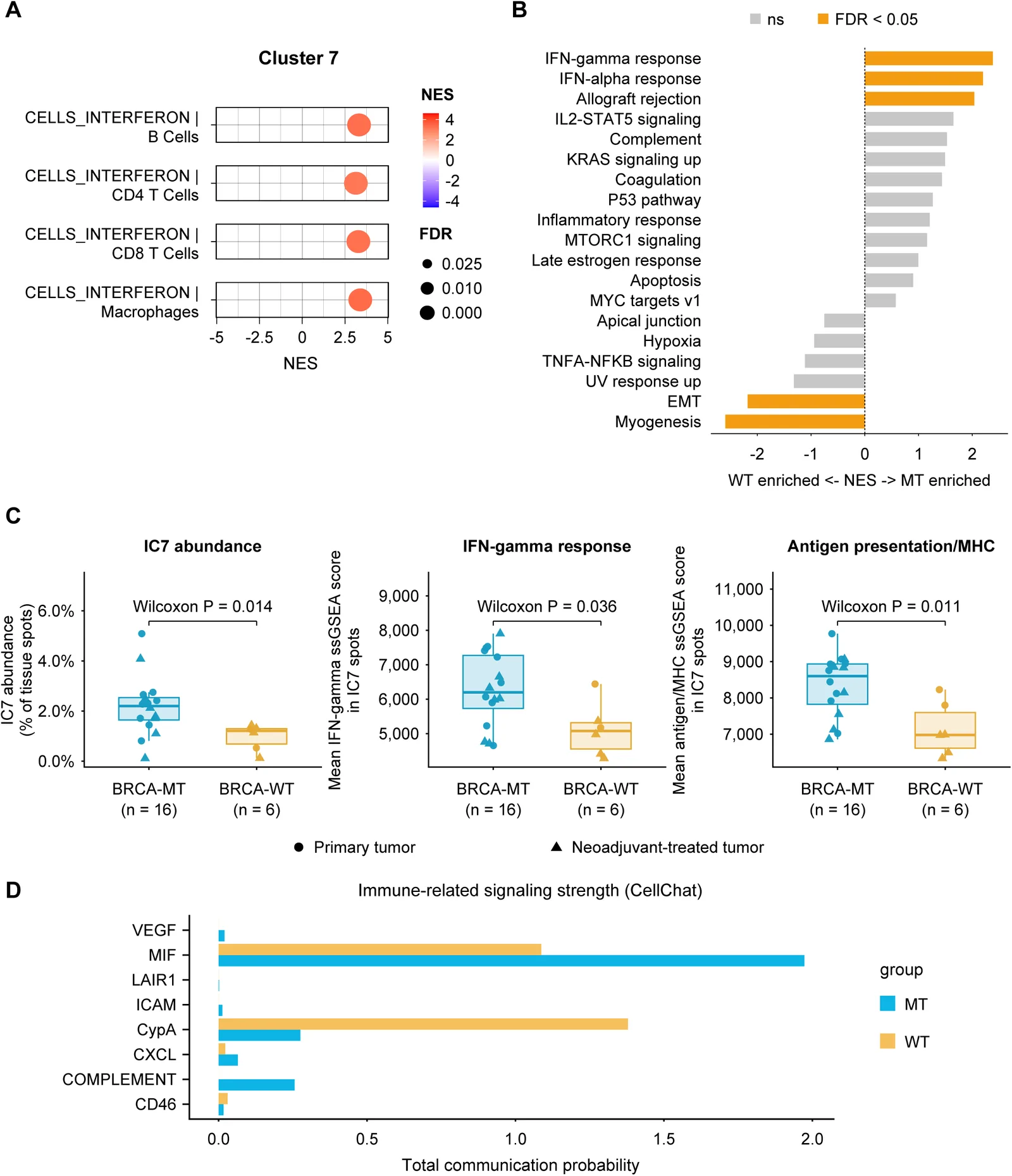
IC7 represents a BRCA-dependent immune-activated spatial region in HGSOC. (A) GSEA of IC7 across the integrated cohort against curated immune signatures, showing activation of interferon (IFN-α/γ) responses, complement signaling, and inflammatory programs. Normalized enrichment scores (NES) are shown for pathways significant at FDR < 0.05. (B) Genotype-stratified Hallmark GSEA comparing IC7 between BRCA-mutant (BRCA-MT) and BRCA-wild-type (BRCA-WT) tumors. Bars show NES (MT vs WT); colored bars indicate FDR < 0.05, gray indicates non-significant. (C) Patient-level comparison of three pre-specified primary immune metrics within IC7 between BRCA-MT and BRCA-WT tumors: IC7 abundance (fraction of tissue spots), IFN-gamma signature score and antigen-presentation/MHC signature score. Each point is one patient; boxes show median and interquartile range. Two-sided Wilcoxon rank-sum p-values are shown (p = 0.014, 0.036, and 0.011). (D) IC7-specific intercellular communication inferred with CellChat from pooled genotype-level BRCA-MT and BRCA-WT datasets, showing total predicted pathway-level communication probability of immune-related pathways.

To determine whether the immune-associated transcriptional program within IC7 reflected differences in cellular composition rather than elevated immune gene signatures alone, we applied RCTD deconvolution with the previously described reference to independently estimate cell-type composition and summarized the results at the patient level (n = 22). IC7 regions showed a higher inferred T/NK plus dendritic-cell fraction than non-IC7 regions within the same patients (median 7.18% versus 4.85%; paired Wilcoxon signed-rank test, p = 2.15 x 10^-4; BH-FDR q = 8.81 x 10^-4; S5B Fig).

To complement the spot-level enrichment, we also assessed patient-level summary values (Fig 7C). IC7 occupied a larger fraction of spatial capture spots in BRCA-MT than in BRCA-WT tumors and IC7 regions in BRCA-MT tumors exhibited higher antigen-presentation/MHC signature scores and higher IFN-gamma signature scores (Fig 7C). All three differences remained significant after Benjamini-Hochberg correction and were unchanged when each retained section was weighted equally within a patient (S8 Fig), indicating that the BRCA-associated immune activation within IC7 is consistent at the patient level. The plotted patient-level values, section-level inputs, and alternative section-weighting analyses are provided in S4 Table.

Because our cohort comprised both treatment-naive and neoadjuvant-treated tumors, we next assessed whether these differences could be attributable to treatment rather than BRCA status. In linear models including both BRCA status and treatment as covariates, the BRCA-associated effect on IC7 remained significant after adjustment for treatment for all three metrics (IC7 abundance, p = 0.044; antigen presentation/MHC, p = 0.011; IFN-gamma, p = 0.044; S4 Table). In these models, treatment status was independently associated with the antigen-presentation/MHC score (p = 0.048) but not with IC7 abundance (p = 0.49) or IFN-gamma activity (p = 0.21); notably, the BRCA effect on antigen presentation persisted even after accounting for this treatment association. The BRCA-MT > BRCA-WT ordering was further preserved within both the treatment-naive subset (n = 9 BRCA-MT, 2 BRCA-WT) and the neoadjuvant-treated subset (n = 7 BRCA-MT, 4 BRCA-WT), although the small size of these subgroups, particularly the two treatment-naive BRCA-WT tumors, precluded statistical significance, and these stratified comparisons should be regarded as exploratory. Thus, we conclude that BRCA-associated immune activation within IC7 is unlikely to be confounded by treatment status.

To assess whether the patient-level IC7 signal was dominated by a single BRCA alteration type, we stratified BRCA-MT tumors by alteration type as a descriptive sensitivity analysis. Germline *BRCA1* (n = 9), germline *BRCA2* (n = 2), *BRCA1* promoter hypermethylation (n = 4), and somatic *BRCA1* (n = 1) tumors all showed higher median IC7 abundance, antigen-presentation/MHC activity, and IFN-gamma activity than BRCA-WT tumors (S6 Fig). Nonetheless, the elevated signal was not confined to any single alteration class, making it less likely that the association was driven exclusively by a single alteration type. This interpretation is consistent with prior work indicating that germline mutation, somatic mutation and promoter methylation of BRCA alterations converge on a common immune-activated phenotype and on a shared homologous recombination–deficient state [22–25].

We next examined whether BRCA-dependent transcriptional differences were accompanied by coordinated changes in predicted intercellular communication patterns. Using CellChat on our pooled genotype-level datasets [20], we compared immune-related signaling programs between BRCA-MT and BRCA-WT tumors. Exploratory CellChat analysis suggested possible differences in predicted signaling activity across selected immune-associated pathways, including MIF, COMPLEMENT, and ICAM (Fig 7D). Predicted activity across these pathways appeared lower in BRCA-WT tumors than in BRCA-MT tumors. Although IC7 was detected in both genotypes, the predicted signaling patterns were broadly concordant with the transcriptomic differences observed within IC7, including greater enrichment of immune-related programs in BRCA-MT and lower enrichment of these programs in BRCA-WT.

To determine whether BRCA-associated differences in immune signaling reflect changes in spatial tissue architecture, we performed join count analysis [26] to compare spatial dependencies of ICs within BRCA-MT and BRCA-WT tumors. This analysis did not reveal significant differences in global spatial patterns between the two groups, indicating that the observed differences in immune signaling are not driven by alterations in spatial organization of the tumors (S7 Fig). Instead, these findings suggest that BRCA status primarily modulates the functional state and communication of immune niches within a conserved spatial framework.

## Discussion

High-grade serous ovarian cancer (HGSOC) is characterized by profound genomic instability, widespread pathway dysregulation, and marked inter- and intra-tumoral heterogeneity. These features define a complex disease biology and contribute to variation across tumors. In this study, our analyses demonstrate that BRCA-MT tumors exhibited increased genomic instability relative to BRCA-WT tumors. A subset of BRCA-MT tumors also displayed relatively elevated predicted neoantigen counts, consistent with the possibility that HRD-associated genomic disruption may enhance antigenic potential in part of the cohort. However, as has been highlighted in ovarian cancer, the presence of predicted neoantigens does not necessarily translate into an effective antitumor immune response, underscoring the complexity of neoantigen biology in this disease [27]. Together with prior evidence linking *BRCA1/2*-associated HRD to chromosomal instability and enhanced tumor–immune interactions, these findings support a model in which BRCA-MT tumors may adopt a more immune-reactive phenotype [4,28,29]. Despite these features, clinical responses remain heterogeneous in HGSOC. Increasing evidence suggests that intratumoral clonal heterogeneity is associated with poorer survival following platinum-based chemotherapy and may underlie therapeutic resistance [30]. Moreover, recent studies indicate that resistant subclonal populations may pre-exist within tumors, potentially limiting the durability of PARP inhibitor responses [31].

At the transcriptional level, bulk RNA sequencing revealed distinct genotype-associated programs. BRCA-MT tumors were enriched for proliferative and DNA damage-associated pathways, including E2F targets, MYC signaling, and G2/M checkpoint activation, whereas BRCA-WT tumors preferentially exhibited transcriptional programs linked to epithelial-mesenchymal transition, hypoxia, and stromal remodeling. These patterns are consistent with previously described proliferative and mesenchymal ovarian cancer subtypes with distinct biological and clinical behaviors [7]. However, bulk transcriptomic analyses struggle to determine if these patterns are clonal or heterogeneous, nor can they resolve how these programs are organized within the tumor and immune microenvironment.

Visium spatial transcriptomics allowed us to investigate a genotype-resolved view of tumor and immune compartmental organization within HGSOC. While recurrent tumor, stromal, and immune compartments were preserved across tumors irrespective of BRCA genotype, indicative of a conserved architectural scaffold, the functional state of immune niches differed markedly between BRCA genotypes. In particular, we identified an immune-enriched spatial cluster (IC7) characterized by interferon signaling, complement activation, and antigen-presentation machinery, with significantly greater activation in BRCA-MT tumors compared with BRCA-WT tumors. Reference-based RCTD deconvolution further supported enrichment of T/NK and dendritic cells within IC7 relative to non-IC7 regions, while showing no significant increase in macrophage or total immune content. Consistent with these observations, recent reports have also indicated that BRCA deficiency may enhance tumor immunogenicity through increased expression of antigen-presentation machinery and inflammatory signaling pathways in ovarian precancerous lesions [32]. Other studies have demonstrated that malignant subclones with distinct copy number profiles can associate with distinct immune and stromal neighborhoods, supporting a model in which genotypic diversification is embedded within, and potentially shaped by, spatial microenvironments [33]. Our data extend these observations by suggesting that genotype-associated immune activation is not uniformly distributed across the tumor but is instead concentrated within discrete immune-permissive niches. While we did not perform a survival analysis on our cohort due to the lack of censoring events, prior studies have demonstrated that the spatial organization and proximity of immune cells, rather than their abundance alone, are critical determinants of clinical outcome in ovarian cancer [34].

In addition to this immune-activated region, we identified a macrophage-rich spatial cluster (IC4) that displays transcriptional features consistent with lipid-associated macrophages (LAMs) [18,35]. LAMs were initially described in metabolic disease and are characterized by expression of specific genes such as *APOE*, *APOC1*, and *GPNMB* [18]. Cross-tissue single-cell integration has since supported the existence of conserved tumor-associated macrophage states across cancers, including macrophage programs enriched in lipid metabolism and immunoregulatory pathways [36]. In breast cancer, LAMs have been further characterized as a distinct immunosuppressive macrophage state that can be actively induced by tumor-associated stromal signals [37]. Although direct stromal–macrophage coupling has been less clearly defined in ovarian cancer, desmoplastic fibroblasts in HGSC upregulate CXCL12 and are associated with immune-enriched stromal regions, supporting a role for fibroblast-derived chemokine signaling in organizing the local immune microenvironment [38]. Crosstalk between CAFs and tumor-associated macrophages (TAMs) through ligand–receptor interactions has been further described across solid tumors [39]. Our pooled expression-based CellChat analysis suggests a potential interaction between a CAF-like cluster and a LAM-like cluster, consistent with models in which chemokine-mediated signaling axes, including CXCL12-CXCR4, contribute to stromal-myeloid crosstalk and immune modulation in breast cancer [37]. Importantly, this LAM-like niche was present across both BRCA genotypes, indicating that macrophage-rich regions represent a conserved structural feature of the HGSOC tumor ecosystem.

Taken together, our findings support a model in which tumor genotype modulates immune activity within a conserved spatial microenvironmental scaffold. In this framework, macrophage-rich stromal niches represent stable structural elements of the tumor ecosystem, while immune activation within defined compartments, such as IC7, is shaped by BRCA-associated genomic instability. This distinction between spatial architecture and functional immune state provides a potential explanation for the diverse immune phenotypes observed across HGSOC tumors. These findings further suggest that effective therapeutic strategies, particularly for BRCA-WT or immune-attenuated tumors, may require combining immune-targeted therapies with approaches that modulate stromal-myeloid interactions, thereby expanding immune-permissive niches within the tumor microenvironment.

These findings should be interpreted in the context of several limitations. First, the cohort is modest in size and unbalanced between groups (genomic cohort, 17 BRCA-MT and 8 BRCA-WT; integrated spatial cohort, 16 BRCA-MT and 6 BRCA-WT), and the study was designed as a retrospective, sample-availability-driven exploratory analysis. We therefore did not perform a formal power calculation; instead, we report effect estimates alongside p-values and treat the immune-deconvolution and cell-cell-communication results as hypothesis-generating. Additionally, 10x Visium spots capture mixed-cell transcriptomes and the integrated clusters should be interpreted as marker-enriched spatio-transcriptional regions rather than purified cell populations. Future experiments examining single-cell transcriptomes and proteomes are needed to further validate these findings and determine the exact cellular compositions of ICs. Similarly, mechanistic experiments would help validate the predicted intracellular communication inferred from expression data. Future work will also benefit from a larger, clinically annotated, and alteration-stratified cohort to allow correlation with clinical outcomes.

Overall, our study highlights that BRCA mutation status is associated with immune-related transcriptional differences within conserved spatial transcriptional regions in HGSOC. As such, these findings provide a spatially aware framework for understanding genotype-associated immune heterogeneity in HGSOC and offer a promising direction for future mechanistic and clinical validation.

## Materials and Methods

### Ethics Statement

The study was approved by the University of Southern California Keck School of Medicine Gynecological Tissue and Fluid Repositories (GTFR) under a protocol approved by the Institutional Review Board (#HS-18–00948). Patients provided written informed consent for sample collection.

### Characteristics of clinical samples

The study was approved by the University of Southern California Keck School of Medicine Gynecological Tissue and Fluid Repositories (GTFR) under a protocol approved by the Institutional Review Board (#HS-18–00948). Patients provided written informed consent for sample collection. We obtained OCT-embedded fresh-frozen blocks from 25 EOC cases and matched buffy coat or normal tissue. The histology of all tumors was confirmed as HGSOC by pathologist review. All tumors were late stage, and the cohort was evenly split between treatment-naïve tumors and tumors that had received neo-adjuvant treatment prior to debulking surgery. We had prior knowledge of germline *BRCA1/2* status from clinical testing reports. All samples were reassessed for germline and somatic variants as described subsequently in Methods. We confirmed known BRCA genotypes and identified an additional sample with *BRCA1* somatic mutation. We additionally assessed *BRCA1/2* promoter methylation as described below and identified four samples with *BRCA1* promoter hypermethylation. Therefore, we stratified our cohort as follows. 10 tumors harboring germline *BRCA1* mutations, 4 tumors with *BRCA1* promoter hypermethylation, 1 tumor with *BRCA1* somatic mutation, and 2 tumors harboring germline *BRCA2* mutations were classified as BRCA-MT. An additional 8 tumors with no alterations in the *BRCA1/2* genes were classified as BRCA-WT. Sample information is summarized in S1 Table.

### RNA and DNA isolation

Genomic DNA and RNA was extracted from fresh frozen OCT-embedded tissue using the Qiagen AllPrep Mini kit (Qiagen, Hilden, Germany) based on the manufacturer’s recommendations. DNA and RNA were quality assessed and quantified using the Qubit Fluorometer (Thermo Fisher Scientific, Waltham, MA) and the Genomic DNA and RNA ScreenTape assays on the Agilent 4200 TapeStation System (Agilent Technologies, Santa Clara, CA). DNA samples were stored at −20 °C and RNA samples at −80 °C until further processing.

### Whole exome sequencing

Whole-exome sequencing (WES) was performed on genomic DNA (50–200 ng) extracted from HGSOC tumor tissue and matched buffy coat or normal tissue. DNA was sheared in 50 µL low-EDTA TE buffer using a Covaris E220 system to generate fragments with an average size of 150–200 bp. Sequencing libraries were prepared using the SureSelect XT Low Input Library Preparation Kit (Agilent) with unique dual indices. Tumor libraries were enriched using a custom SureSelect V6 + UTR + OneSeq LowRes capture probe set (Agilent), while germline libraries were enriched using the SureSelect Human All Exon V6 capture kit supplemented with custom probes. Libraries underwent initial quality control sequencing on the Illumina MiSeq platform, followed by pooled sequencing on the Illumina NovaSeq 6000 using a 300-cycle kit. Sequencing metrics can be found in S2 Table.

### Total RNA library preparation and sequencing

RNA integrity was assessed using the High Sensitivity RNA ScreenTape assay on the Agilent TapeStation system. Whole transcriptome RNA sequencing libraries were prepared at the USC Norris Molecular Genomics Core using the SMARTer Stranded Total RNA-Seq Kit v3 (Takara Bio). Final libraries were evaluated for fragment size and quality using the Agilent D1000 ScreenTape assay and sequenced on an Illumina NextSeq 2000 P3 flow cell with paired-end 2 × 100 bp reads. FASTQ files are merged and aligned to GRCh38 (Ensembl) by STAR (v2.6.1d) [40]. Picard MarkDuplicates (v2.18.22) (https://broadinstitute.github.io/picard/) identified duplicate reads in the aligned BAM files, producing marked bams and metrics files. Salmon (v0.11.3) [41] was used to quantify transcript abundance against the GRCh38 reference transcriptome. Gene-level read counts were generated using featureCounts (Subread v2.0.0) [42] with GENCODE v29 primary assembly annotation. SNPs and indels were identified using GATK HaplotypeCaller [43]. RNA sequencing quality metrics were assessed using Picard tools (https://broadinstitute.github.io/picard/), and alignment statistics were generated with SAMtools [44]. Sequencing metrics can be found in S2 Table.

### Germline variants analysis

Germline variants were identified from normal sample BAM files using FreeBayes (v1.3.6) [45] and Strelka2 (v2.9.2) [46] with the GRCh38 reference genome. Variant call format (VCF) files were annotated using SnpEff (v5.1d) [47] and filtered using SnpSift (v5.1d) [48]. Variants with allele frequencies exceeding 3% in population databases were excluded, and only pathogenic or likely pathogenic variants were retained. High-confidence germline variants were defined as those detected concordantly by both variant callers.

### EPIC DNA methylation analysis

DNA methylation profiling was performed using the Illumina Infinium MethylationEPIC BeadArray (EPIC) at the USC Norris Molecular Genomics Core Facility. Bisulfite-converted DNA was whole-genome amplified, enzymatically fragmented, and hybridized to the EPIC BeadArray according to the manufacturer’s protocol. Arrays were scanned using the Illumina iScan system to generate raw intensity data (*.IDAT files). Raw data were processed in R using the minfi package (v1.50.0). Probes with detection p-values >0.05, non-specific probes, and probes located on the sex chromosomes were removed. DNA methylation levels were represented as β-values calculated as M/(M + U), where M and U represent methylated and unmethylated probe intensities, respectively.

### Somatic variant calling and mutational signature analysis

Raw sequencing data were demultiplexed using bcl2fastq (v2.20.0.422), and reads were aligned to the GRCh38 reference genome using BWA-MEM (v0.7.17) [49]. Somatic single nucleotide variants (SNVs) and small insertions/deletions were identified using GATK (v4.1.0.0) Mutect2 [50] following GATK best practices. Variants were annotated using the Ensembl Variant Effect Predictor (VEP) [51]. Tumor mutational burden (TMB) was calculated as the number of nonsynonymous somatic mutations per coding megabase. Somatic mutational signatures were extracted using SigProfiler [52] based on the 96-channel base substitution framework and compared against COSMIC v3 [11] reference signatures. Mutation annotation format (MAF) files were further processed and visualized using maftools [53].

### Bulk copy number analysis

Allele-specific copy number analysis was performed using Sequenza(v3.0.0) [54] on paired tumor–normal BAM files aligned to the GRCh38 reference genome. A genome-wide wiggle track was first generated using a 50-bp window size. Sequenza then quantified tumor and normal read counts within each genomic bin and applied segmentation to identify regions with copy number alterations. The outputs were used to visualize genome-wide copy number profiles, read depth ratios, and B-allele frequencies.

### Homologous recombination deficiency (HRD) analysis

Homologous recombination deficiency (HRD) was quantified using scarHRD [13]. Loss of heterozygosity (LOH), telomeric allelic imbalance (TAI), and large-scale state transitions (LST) were computed from somatic WES data as genomic scar metrics. The HRD score was defined as the sum of LOH, TAI, and LST, with scores ≥42 classified as HRD-positive [14]. The scarHRD-derived genomic scar metrics (LOH, TAI, and LST) and the scarHRD score for each sample are provided in S3 Table.

### Differential gene expression analysis

Differential gene expression (DGE) analysis between BRCA-MT and BRCA-WT tumors was performed using the DESeq2 R package (v1.44.0) [55]. Log2 fold changes (log2FC) were calculated for each gene, and statistical significance was assessed using Wald tests. To control for multiple hypothesis testing, p-values were adjusted using the Benjamini–Hochberg procedure, with a false discovery rate (FDR) threshold of 0.05 considered statistically significant.

### Pathway enrichment analysis

Gene set enrichment analysis (GSEA) was performed to identify biological pathways differentially represented between BRCA-MT and BRCA-WT tumors. For bulk RNA seq analysis, ranked gene lists were generated based on log2 fold change values from DESeq2 output and submitted to pre-ranked GSEA against the Hallmark gene sets [56] from the Molecular Signatures Database (MSigDB) [57]. Normalized enrichment scores (NES) were assessed to determine enrichment between groups. Pathways with FDR-adjusted q-values < 0.05 were considered significantly enriched. For application to spatial transcriptomics data, single-sample GSEA was applied to the spatial expression data at the level of each spatial feature. The sample normalization method was set to rank, with a weighting exponent of 0.75 and a minimum gene-set overlap of 10 genes. To apply GSEA at the cluster level, differentially expressed cluster markers were first determined by Wilcoxon rank sum testing, using Seurat FindAllMarkers feature. Gene lists pertaining to each cluster were analyzed by preranked GSEA against Hallmark gene sets [56] and curated immune signatures [19].

### Immune cell enrichment analysis (xCell)

xCell [58] was used for descriptive annotation of macrophage-related enrichment patterns across integrated spatial clusters. Normalized expression profiles summarized by integrated cluster were submitted to xCell using default parameters, and macrophage-related enrichment scores were summarized across integrated clusters to support spatial-cluster annotation.

### Prediction of somatic neoantigens

Patient-specific HLA class I and class II genotypes were inferred from WES data using HLA-LA [59]. Somatic SNVs and INDELs identified by Mutect2 (as described above) were filtered and prepared for neoantigen prediction following the pVACseq input preparation guidelines. Tumor-specific neoantigens prediction was performed using pVACseq from the pVACtools suite [60]. Candidate neoantigens were retained if they demonstrated predicted strong binding to patient-specific HLA alleles (IC50 < 500 nM) and transcript expression > 1 TPM. RNA-level variant allele expression was further confirmed using bam-read count on paired RNA-seq BAM files. The final number of high-confidence expressed neoantigens per sample was used for downstream analyses.

### Visium spatial gene expression assay and sequencing

Two non-adjacent tissue sections were randomly selected from each of 24 fresh-frozen, OCT-embedded HGSOC tumors (n = 48 sections). The sections were cryosectioned at 10 µm thickness and mounted onto 10x Genomics Visium Spatial Gene Expression slides. Sections were H&E-stained and imaged using a Zeiss Axio Imager.M2 at 10 × magnification. Tissue permeabilization conditions were optimized using the Visium Spatial Tissue Optimization Kit, with a final incubation time of 30 minutes. Spatially barcoded cDNA was synthesized in situ and processed for library construction according to the manufacturer’s protocol. Libraries were sequenced on an Illumina NovaSeq 6000 with paired-end 2 × 100 bp reads to exceed the manufacturer’s recommended minimum depth of 50k read pairs per spot covered with tissue.

### Spatial transcriptomic data processing and normalization

Raw sequencing data were processed using Space Ranger (v1.1.0, 10x Genomics). Demultiplexed FASTQ files were aligned to the GRCh38 reference genome, and UMI-based gene expression matrices were generated. To assess data quality, we evaluated median genes and median UMI counts per spot in each section. Sections were retained if the median number of genes per spot was ≥ 1,500 and the median number of UMI per spot was ≥ 2500. Seven sections fell below this threshold and were excluded from further analysis. These are identified in S2 Table, along with full QC metrics per section. Spatial feature matrices were imported into Seurat (v4.3.3) [61], where spatial features refer to Visium capture spots. The final integrated analysis retained 40,002 spatial features from 39 tissue sections across 22 patients. The raw Spatial assay contained 36,601 gene features and 492,644,581 total UMI counts, with a median of 10,128 UMIs and 3,723 detected genes per spatial feature. Expression matrices from individual sections were normalized using SCTransform (v0.3.5) [62]. All downstream analyses were performed on SCTransform-normalized or integrated expression values, as appropriate for each analysis.

### ESTIMATE analysis

Tumor purity and stromal and immune content were inferred using the ESTIMATE R package (v1.0.13) [63]. Stromal scores, immune scores, and tumor purity estimates were computed for each spatial feature and visualized to assess spatial heterogeneity in tumor composition.

### Dimensionality reduction and clustering

Principal component analysis was performed on SCTransform-normalized data [62] using the top 30 principal components. Cluster resolution was selected based on stability metrics, ESTIMATE-derived tumor purity, UMAP structure, and spatial localization patterns. Two-dimensional embeddings were generated using UMAP.

### Cohort integration

Spatial feature matrices from individual tissue sections were normalized using SCTransform (v0.3.5) [62] prior to integration. Shared features across samples were identified and used to compute sample-specific principal components (top 30 PCs). Integration anchors were then identified using the reciprocal PCA (RPCA) method in Seurat (FindIntegrationAnchors with reduction = “rpca”) [61,64], and datasets were harmonized using IntegrateData to produce an integrated expression matrix. Downstream dimensionality reduction and clustering were performed on the integrated data using RunPCA, FindNeighbors, FindClusters, and UMAP.

### Patient-level statistics

For patient-level comparisons of IC7, all retained Visium sections from each patient were pooled before calculating one value for each of the three patient-level outcomes: IC7 abundance, IFN-gamma activity, and antigen-presentation/MHC activity. IC7 abundance was defined as the total number of IC7 spots divided by the total number of tissue-covered spots across all retained sections from the same patient. IFN-gamma activity was defined as the arithmetic mean of the per-spot ssGSEA scores for the Hallmark Interferon Gamma Response gene set (HALLMARK_INTERFERON_GAMMA_RESPONSE) across all IC7 spots from the same patient. Antigen-presentation/MHC activity was calculated using the same aggregation procedure with the BioCarta MHC Pathway gene set (BIOCARTA_MHC_PATHWAY). BRCA-MT and BRCA-WT tumors were compared using two-sided Wilcoxon rank-sum tests with the normal approximation and continuity correction. Benjamini-Hochberg correction was applied across the three prespecified primary comparisons. As an aggregation-sensitivity analysis, each metric was first calculated separately within each retained section, after which an alternative patient-level value was calculated as the unweighted arithmetic mean of the section-level values. The same Wilcoxon tests and Benjamini-Hochberg correction were applied to these alternative patient-level values.

To assess potential confounding by treatment, each primary pooled patient-level metric was additionally modeled as a function of BRCA status and treatment status using a linear model. Within treatment strata, BRCA-MT and BRCA-WT tumors were summarized descriptively using the number of patients, median, mean, and direction of difference for each metric. Patient-level values, section-level inputs, aggregation-sensitivity results, and treatment-adjusted analyses are provided in S4 Table.

### Reference-based spatial deconvolution (RCTD)

We performed reference-based spatial deconvolution using RCTD in the spacexr R package (v2.2.1) [65]. We constructed the deconvolution reference from the public HGSOC single-cell RNA-seq reference (GSE165897) [17], collapsing the cell types into broad cell classes, including tumor/epithelial, CAF, endothelial, B cell, T cell, NK cell, dendritic cell, macrophage, mast cell, and mesothelial categories. Then, we input the collapsed raw counts to the Reference function using a maximum of 1,000 cells per class, randomly selected with a fixed seed (seed = 1). The final reference comprised 8,883 cells, including 1,000 cells in each class, except for mast cells (n = 804) and endothelial cells (n = 79). RCTD was run separately for each retained Visium section using the capped reference, raw Spatial-assay counts, total spot UMI counts, spot coordinates and the default parameters of *create.*RCTD and *run.*RCTD functions from the spacexr R package. Cell-type weights were normalized per spot and merged with integrated cluster, patient, and BRCA-status metadata. IC7 versus non-IC7 and IC4 versus non-IC4 regions were compared at the patient level using two-sided paired Wilcoxon signed-rank tests, with Benjamini-Hochberg correction across evaluated IC-by-metric RCTD comparisons. The combined T/NK plus dendritic-cell fraction was defined as the sum of broad T-cell, NK-cell, and dendritic-cell fractions. BRCA-MT and BRCA-WT tumors were compared within IC7 using Wilcoxon rank-sum tests as an exploratory genotype-stratified analysis.

### Cell–cell communication analysis (CellChat)

To explore predicted ligand-receptor relationships among spatially defined clusters, we applied CellChat (v2.2.0) [20]. Normalized expression matrices were used as input, and cluster identities were defined by integrated spatial clustering (IC1-IC7). For the integrated cohort analysis, spatial features were pooled across sections and grouped by integrated cluster identity. Overexpressed genes and ligand–receptor interactions were identified using identifyOverExpressedGenes and identifyOverExpressedInteractions. Communication probabilities were estimated using computeCommunProb with the trimean estimator (population.size = FALSE, 100 permutations, seed = 1), followed by computeCommunProbPathway and aggregateNet. For the genotype-level analysis, CellChat was run separately on pooled BRCA-MT and BRCA-WT spatial features using integrated-cluster identity as the grouping variable. For each pathway, total predicted communication probability was calculated by summing pathway-level probabilities (netP$prob) across all source–target cluster pairs within each genotype-level network.

### Join count statistics for spatial adjacency analysis

Rasterization and join count analysis were performed using the stJoincount Bioconductor package (version 1.10.0) [26], which implements spatial adjacency testing for 10x Genomics Visium data. Briefly, the method constructs a rectangular raster encompassing the tissue area, with resolution optimized to ensure one barcode per pixel without gaps. A queen-style neighborhood structure (including both orthogonal and diagonal neighbors) was generated. Background pixels and isolated features without neighbors were excluded. Multicategorical join count statistics were computed using the joincount.multi function, which tabulates observed versus expected adjacency counts for all possible cluster pairings under a spatial randomness model. Z-scores were calculated as the standardized difference between observed and expected counts. To account for the absence of multiple-testing correction within the framework, a stringent significance threshold of |z| ≥ 3 (approximately P < 0.00135) was applied.

## Supporting information

S1 Fig Distribution of TP53 mutation types across protein regions. Distribution of TP53 mutation types stratified by protein region, comparing the DNA-binding domain (exons 5–8) with other exons. Bars are colored by variant classification (missense, nonsense, frameshift, splice-site, and other).
(TIF)

S2 Fig Sequencing quality metrics for Visium spatial transcriptomics. Per-section quality metrics across all profiled Visium tissue sections (n = 46), shown as separate panels for (A) total genes detected, (B) sequencing saturation, (C) number of spots under tissue, (D) reads mapped confidently to the genome, (E) reads mapped confidently to the transcriptome, (F) total sequencing read pairs, (G) median genes per spot, and (H) median UMI counts per spot. Each point/bar corresponds to one tissue section.
(TIF)

S3 Fig Dimensionality reduction and per-sample composition of the integrated dataset. (A) UMAP embedding of unintegrated spatial features colored by tissue section (Sample_ID) and (B) by patient (Case_ID) (C) Patient-specific UMAP embeddings for the 17 patients with two retained Visium sections. For each patient, spots from the two sections were analyzed jointly, and PCA and UMAP were recalculated independently using only that patient’s spots. Colors indicate section identity, and each point represents one tissue-covered Visium spot. (D) Number of spatial features assigned to each integrated cluster (IC1–IC17) per tissue section. (E) Relative proportion of each integrated cluster within BRCA-MT and BRCA-WT tumors. Integrated clustering was performed at resolution 0.3.
(TIF)

S4 Fig Additional annotation of integrated clusters (IC1–IC17). (A) Violin plots of tumor purity, stromal score, and immune score across all 17 integrated clusters, inferred using ESTIMATE. (B) Cluster-level gene set enrichment analysis (GSEA) against Hallmark gene sets for integrated clusters, grouped by functional category (development, immune, metabolism, oncogenic, and stress response). Dot size represents –log10(FDR) and color indicates normalized enrichment score (NES); only pathways with FDR < 0.05 are shown.
(TIF)

S5 Fig Patient-level ssGSEA ranking and reference-based RCTD deconvolution of selected spatial transcriptional regions. (A) Patient-level ssGSEA scores for signatures in which IC7 ranked highest among shared integrated clusters, including IFN-gamma response, IFN-α response, and TNFα/NF-κB signaling. Boxplots show patient-level scores across integrated clusters, with IC7 highlighted in red.(B) Patient-level paired comparison of the summed RCTD-inferred broad T-cell, NK-cell, and dendritic-cell (T/NK + DC) fraction in IC7 versus non-IC7 regions. Each point represents the mean RCTD fraction across spots within the indicated region for one patient; lines connect paired values from the same patient. Horizontal bars denote across-patient medians. (C) Patient-level paired comparison of the RCTD-inferred macrophage fraction in IC4 versus non-IC4 regions, plotted as in (B).
(TIF)

S6 Fig Patient-level IC7 metrics across BRCA alteration subtypes. (A) IC7 abundance, (B) mean HALLMARK_INTERFERON_GAMMA_RESPONSE ssGSEA score across IC7 spots, and (C) mean BIOCARTA_MHC_PATHWAY ssGSEA score across IC7 spots in tumors with germline BRCA1 (n = 9), germline BRCA2 (n = 2), BRCA1 promoter hypermethylation (n = 4), somatic BRCA1 (n = 1), or BRCA-WT status (n = 6). Each point represents one patient, point shape denotes primary or neoadjuvant-treated tumor, and horizontal bars indicate subgroup medians. Dashed lines separate the BRCA alteration subtypes from BRCA-WT tumors.
(TIF)

S7 Fig Spatial dependency analysis of integrated clusters by BRCA status. (A) Representative spatial cluster maps and per-section IC–IC spatial co-occurrence (join count) matrices for two tumors (HR5, HR10). (B) Join count Z-scores for IC pairs involving IC1–IC7, compared between BRCA-MT and BRCA-WT tumors; boxes show the distribution across patients within each group. Positive Z-scores indicate spatial attraction and negative Z-scores indicate spatial avoidance between cluster pairs. (C) Representative spatial maps and UMAP embeddings highlighting the IC3–IC4 (CAF–macrophage) and IC1–IC7 (tumor–immune) spatial relationships in selected tumors.
(TIF)

S8 Fig Sensitivity of patient-level IC7 metrics to section aggregation. (A-C) Comparison of primary pooled patient-level values (x-axis) and unweighted means of section-level values (y-axis) for (A) IC7 abundance, (B) HALLMARK_INTERFERON_GAMMA_RESPONSE ssGSEA scores, and (C) BIOCARTA_MHC_PATHWAY ssGSEA scores.Each point represents one patient and is colored by BRCA status. Filled and open symbols indicate patients with two retained sections (n = 17) and one retained section (n = 5); values for single-section patients are identical by definition. Dashed lines indicate identity.
(TIF)

S1 Table Case demographics and clinical annotation. (XLSX)

S2 Table Sequencing and quality control metrics for whole exome sequencing, RNA sequencing, and Visium spatial transcriptomics. (XLSX)

S3 Table ScarHRD score and predicted neoantigen counts for each sample. (XLSX)

S4 Table Patient-level IC7 metrics, section-level inputs, aggregation-sensitivity analysis, and treatment-adjusted models. (XLSX)
